# Observations on Dr. Taylor’s Paper on Flagellation of the Abdomen, Etc.

**Published:** 1880-04

**Authors:** Rufus W. Griswold

**Affiliations:** President of the Hartford Co. (Conn.) Medical Association, Rocky Hill, Conn.


ARTICLE II.

### OBSERVATIONS ON DR. TAYLOR'S PAPER ON FLAGELLATION OF THE ABDOMEN AND OF THE BACK OF THE INFANT, IN RELATION TO POST-PARTUM HÆMORRHAGE.

BY RUFUS W. GRISWOLD, M. D.

President of the Hartford Co. (Conn.) Medical Association, Rocky Hill, Conn.

The valuable paper of Prof. Isaac E. Taylor, in THE INDEPENDENT PRACTITIONER for February, stimulates kindly comment and criticism. The procedure of flagellation of the abdomen as a means of arresting post-partum hæmorrhage will be new to most members of the profession, as will also the like process of flagellation of the back of the child as a preventive. For the practical suggestions given us, thousands of physicians will thank Dr. T., although they may not all be in exact accord with him in all the points of his article. As the personal experience of even the humblest of the profession may have in it something of value upon questions of different treatment in given circumstances, I venture to offer a few thoughts upon some parts of the doctor's paper.

Upon the matter of spanking the back of the half-born child, as a means, by the reflex irritative action to be derived from it upon the contractility of the uterus, to prevent bleeding, one to whom the idea is entirely new, and who has never made use of it, is not in the position to say anything unfavorable; particularly when, theoretically, the plan commends itself to acceptance. But one may venture to observe, that the few cases of seemingly, and possibly real, benefit from the spanking, cited by Dr. T., are not sufficient to establish the theory as a fact. Few obstetricians of much experience can have failed to observe that where there has been severe hæmorrhage in one or more deliveries, succeeding ones may offer no more, and perhaps even less than the usual amount of bleeding, even when there have been no special steps taken in the way of prevention. It is not an infrequent thing for the physician to be told, at the lying-in bedside, that the lady at her last or some former delivery flowed badly. It is well to be informed of this: knowing it, one is more inclined to be on the watch for trouble, and perhaps be better prepared for it if it comes. My personal experience (which covers nearly thirty years in practice) is that it does not often come when looked for; indeed, though I can remember having been frequently put on guard against this possible danger, I do not now recollect a single case of this kind in which I have had hæmorrhage; it comes, as a rule, when you do not expect it. But if, in the cases where one had been informed of flooding on former occasions, he had always adopted the procedure of flagellation, or any other special procedure, and had not had the flooding, his first, and perhaps legitimate, though fallacious, conclusion would be that the flagellation, or other special procedure, deserved the merit of salvation. It is not a perfectly reliable basis upon which to argue effect, that because a man who was ailing last night took a pill and is well this morning, that it was the pill that cured him; nor indeed from a dozen such coincidences, since it is every day happening that another dozen men, ailing similarly, were well the next morning, not having taken any pill at all. So also, when a physician, having taken some particular action in the way of prevention against flooding in the cases of certain women who had flowed badly at some previous times, and is seemingly successful in his treatment, he may find reason to doubt the especial efficacy of it, if he knows that as many or more other women who had had flooding at former deliveries, have gone through another one without that trouble, and without the same or any other special means having been used to prevent it. Nearly every case of confinement, in *some* of the phenomena which it presents, stands alone, independently of anything that may have been done or taken in the case. In the same woman one labor is easy and next severe,—one is short, the next is protracted,—one requires interference, and the next does not,—one is accompanied with alarming hæmorrhage, and the next is below the average of bleeding; and this without anything having been done to hinder or to help. But if anything has been done, the more satisfactory issue is credited to the virtue of the Has Been—a credit that involves the same species of illogical logic that is the illusory foundation of the supposed virtue of the incomprehensible infinitesimalism of the homœopath. The supposed effect, in the cases related by Dr. Taylor to prove the virtue of flagellation of the back of the child as a preventive of uterine hæmorrhage, and in other similar cases, may be purely supposititious. To bring up the theory to the dignity of an established fact will require long, careful, thorough and widespread observation. To some enthusiast in statistics this point opens up a fine field for long-continued inquiry; meantime, the large body of obstetricians will scarcely enter upon an indiscriminate flagellation of the back of every babe whose mother has previously been unfortunate enough to have had post-partum hæmorrhage.

The other suggestion in Prof. T.'s paper, namely, flagellation of the abdomen to arrest hæmorrhage, and as a substitute for the introduction of the hand into the uterus, which is of much more importance than the first proposition considered, is not so uncertain as regards the relation between procedure and result. When one attacks, with the same mode of treatment, a number of similar cases of palpable and unmistakable uterine flooding, and with unvarying success, there is little room left for doubt of the relation between the treatment and the effect observed. One could not baste the abdomens of very many flooding patients, and find contractility of the uterus almost immediately following, without being convinced that the basting had done good. This part of the doctor's paper is, therefore, very valuable. It shows a mode for the production of sudden shock in cases of post-partum bleeding that will be new to most of us, although it is only another, but in some respects better, mode of the sudden application of cold water to the abdomen to induce the shock. The general principle is the same; the flagellation is a modification of the methods. In the earlier years of my practice, led thereto more especially by something in the lectures of Prof. Gilman, of the New York College of Physicians and Surgeons, I almost invariably *flung* a diaper or napkin, rung out in cold water, upon the abdomen of every lying-in woman under my care—generally immediately after the expulsion of the placenta, but sometimes before. In many cases it was repeated. Many other practitioners do the same in many cases. It was frequently objected to by friends, from the fear that it would induce "cold"; and so I learned to qualify the water with a dash of alcohol or liquor, as a relief to the fears. In later years, perhaps having grown a degree careless, in consequence of familiarity with the obstetric procedure, I have relaxed in the application of this cold cloth. The cloth was not laid on, but *flung*, or *slapped*. The effect was sudden shock, and following contractility of the uterus, and consequent less flooding, and less danger of flooding. It seems to me that this was good practice; it also seems to me, looking back, that the abandonment of it in many cases has left more trouble from bleeding. The application of cold cloths in this way is essentially the same as the flagellation of Dr. Taylor; but it is not exactly it, and would not, in all cases, likely be as good. The basting is a modification, and will undoubtedly prove a valuable modification of the older ways of suddenly applying cold. What would be the effect of *dry* basting is not clearly indicated in Prof. T.'s paper, as he does not detail his experience with a dry towel.

We come now to the point of considering the *kind* of cases in which flagellation of the abdomen is to be substituted for the introduction of the hand to the uterine cavity. Dr. Taylor says: "I refer especially in this paper to uterine atony or inertia produced from any cause, and producing a functional paresis or sopor of the uterus, appearing sometimes before, but more especially after the delivery of the placenta." But the animus of his paper implies something beyond the application of basting to the management of this class of cases, since he quotes approvingly the testimony of Dewees that "he had not found it necessary to introduce the hand for the purpose of stopping uterine hæmorrhage after the placenta was expelled, for thirty-five years, and regarded it as frightfully unnecessary and pernicious": he also gives his own experience, saying, "I have had no occasion to insert my hand into the uterus for the delivery of the placenta except three times in thirty-five years, and then it was for complete adhesion of that organ to the parietes of the uterus." The inference from these statements is logical that, in the opinion of Dr. T., the introduction of the hand into the womb is very seldom necessary, nor indeed justifiable, although he does not advise fully to that degree. We may be allowed here to observe that Dr. T. does not clearly and explicitly enough indicate to us, as we might reasonably expect a man of his experience and professional eminence to do, the class and kind of cases in which introduction of the hand should be preferred, if ever, to flagellation of the abdomen. Now, leaving out of the account any consideration of the management of cases previous to the expulsion of the placenta, let us see if it is not possible to so group the conditions that give us hæmorrhage after the placenta is removed, as to get some intelligent idea of what cases flagellation may be applied to, and what not, as a substitute for the hand *in utero*. Post-partum flooding presents us with two conditions of the uterus of very broad distinction. One is a condition of inertia, or absence of contraction (I leave out here, as Dr. T. does in his paper, all consideration of *diseased* conditions); the other is of abnormal or irregular contraction. Which is the most dangerous of these two it might be difficult to say. The judicious treatment of these two conditions may be quite unlike; while in the first the application of cold, as the first step, might be advisable, in the other it would not. Flagellation, or the cold douche, so far from correcting an irregular contraction, might only—would most likely—intensify it. What is first needed in this condition is the restoration of the cavity of the uterus to its normal ovoid form; and it is matter of doubt if this can with any certainty be safely trusted to any other treatment than the introduction of the hand. Hour-glass contraction, cupola, nile-chamber, or whatever other irregularity may be encountered, should be reduced by a bold but gentle manipulation, and the accumulation of clots generally found in such a uterus be swept out with the retiring hand. If contraction do not follow the restoration of the uterine cavity to a normal shape, then, either before or after the withdrawal of the hand, the cold douche, or perhaps preferably the basting suggested by Dr. T., is the next thing to be done. Again, in flooding from *inertia* of the uterus, we encounter two very different conditions: first, a patent, open, outpouring hæmorrhage; second, and what is worse, a concealed, internal bleeding—the blood remaining in the womb, coagulating into clots, and accumulating till the woman is at the point of death. This condition, vividly delineated by the writers on obstetrics, is the more fearful because it is concealed. Until one has become somewhat acquainted with the disturbances it creates, he may not recognize the mischief that is going on. The books, indeed, tell us that we may diagnose the condition by the feel of the uterus through the walls of the abdomen; but as a matter of fact this is not always true, except it be to those physicians who write books and lecture in the schools of medicine. The only sure road to the determination of the exact state is through the vagina. Having determined the point by carrying the fingers to the mouth of the womb, and made the discovery of a probable pint or quart of clots beyond, there still remains the possible complication of irregular contraction of the fundus with inertia of the rest of the organ. But if we have a loaded uterus purely from inertia, the cavity perfectly normal as to shape, what then? Is it the better practice to consume time in the titillation of the mouth of the womb, or retiring the hand altogether and applying the douche or the flagellation, or some other method, seek to make the womb unload itself; or to adopt the certain, speedy and safe step of emptying it by the hand? Any of the modes in this first order of procedures is a tampering with peril in a moment of peril when there is no moment to spare; the other is an immediate dislodgment of the clots, and along with them generally the forcible expulsion of the hand and the solution of the trouble. But if the hand is not driven from the uterus, or rather if there is not a forcible attempt made by the uterus to drive it out, then the supplementary procedure of flagellation rationally commends itself for approval. In the other condition of inertia, namely, patent, outflowing hæmorrhage, the basting recommended by Dr. T. certainly commends itself to our reason as a first measure to be adopted in preference to and as a substitute for the introduction of the hand.

Now, whether or not I have succeeded in making distinct the differences of condition of hæmorrhagic uteri after delivery of the placenta, so clearly as to make differences of treatment rational, and to show how, in one condition, the employment of the hand may be best, and in another the abdominal basting of Dr. Taylor, I do not know; but as a matter of practical experience I know that these distinctions exist, and they are important ones. Into that flaccid, dish-cloth kind of womb which bleeds, and empties itself as fast as it bleeds, I have never intruded. I have treated them with a seven feet head of water, and so far always with success. Flagellation may be preferable in such cases, and is perhaps less severe; though possibly more likely, if long continued, to produce disagreeable soreness of the muscles of the abdomen. But into the inert uterus which has become loaded with clots I have frequently ventured, always with satisfaction at the results, and never with any following misfortune. Whether or no life has been saved by the procedure may not be certain; but none has been lost. Still, it is well to remember that though one may have attended his five hundredth or his thousandth case without serious result from hæmorrhage, the next to which he is called may present some disagreeable feature that can be better conquered by some new mode of treatment than by the old; and so we may have a chance to remember gratefully the suggestion of abdominal flagellation.

Purposely there has been an avoidance in this paper of any remarks upon any other modes of treatment of uterine hæmorrhage, as being foreign to the particular question of the advisability of substituting for the introduction of the hand the especial mode brought forward by Dr. T. Purposely also there has been an avoidance of citing any authority in favor of the hand *in utero* in cases of flooding. I have also refrained from detailing any cases in illustration of the difficult points of distinction between forms of uterine flooding, and the differing treatments based upon the distinctions made therein, though cases might be given that would emphasize and elucidate what I have endeavored to present. I may hope that it is sufficiently clear without the illustrations.

